# Saving of Time Using a Software-Based versus a Manual Workflow for Toric Intraocular Lens Calculation and Implantation

**DOI:** 10.3390/jcm11102907

**Published:** 2022-05-20

**Authors:** Barbara S. Brunner, Nikolaus Luft, Siegfried G. Priglinger, Mehdi Shajari, Wolfgang J. Mayer, Stefan Kassumeh

**Affiliations:** 1 Department of Ophthalmology, University Hospital, LMU Munich, Mathildenstrasse 8, 80336 Munich, Germany; barbara.brunner@med.uni-muenchen.de (B.S.B.); nikolaus.luft@med.uni-muenchen.de (N.L.); siegfried.priglinger@med.uni-muenchen.de (S.G.P.); mehdi.shajari@med.uni-muenchen.de (M.S.); stefan.kassumeh@med.uni-muenchen.de (S.K.); 2 Department of Ophthalmology, University Hospital, Theodor-Stern-Kai 7, 65933 Frankfurt, Germany

**Keywords:** toric intraocular lens, cataract surgery, cataract surgery workflow, efficiency, refractive surgery, refractive lens exchange, clear lens exchange

## Abstract

Background: To determine whether there is a significant saving of time when using a digital cataract workflow for digital data transfer compared to a manual approach of biometry assessment, data export, intraocular lens calculation, and surgery time. Methods: In total, 48 eyes of 24 patients were divided into two groups: 24 eyes were evaluated using a manual approach, whereas another 24 eyes underwent a full digital lens surgery workflow. The primary variables for comparison between both groups were the overall time as well as several time steps starting at optical biometry acquisition until the end of the surgical lens implantation. Other outcomes, such as toric intraocular lens misalignment, reduction of cylinder, surgically induced astigmatism, prediction error, and distance visual acuity were measured. Results: Overall, the total diagnostic and surgical time was reduced from 1364.1 ± 202.6 s in the manual group to 1125.8 ± 183.2 s in the digital group (*p* < 0.001). The complete time of surgery declined from 756.5 ± 82.3 s to 667.3 ± 56.3 (*p* < 0.0005). Compared to the manual approach of biometric data export and intraocular lens calculation (76.7 ± 12.3 s) as well as the manual export of the reference image to a portable external storage device (26.8 ± 5.5 s), a highly significant saving of time was achieved (*p* < 0.0001). Conclusions: Using a software-based digital approach to toric intraocular lens implantation is convenient, more efficient, and thus more economical than a manual workflow in surgery practice.

## 1. Introduction

The use of toric intraocular lenses (IOL) to correct corneal astigmatism has drastically changed cataract surgery over the last decade and broadened the range of indications for refractive lens exchanges [1]. The prevalence of astigmatism increases with age, with approximately 40–50% of individuals over the age of 60 years presenting a corneal astigmatism of at least 1.00 diopters (D) and thus becoming eligible for astigmatic correction using toric intraocular lenses [2,3]. In these cases, toric intraocular lenses are particularly suited to achieve postoperative independence from glasses as well as increased patient satisfaction. Not surprisingly, the implantation of toric intraocular lenses has gained massive importance in the past few years.

To enable neutralization of corneal astigmatism, a precise preoperative calculation of the toric intraocular lens power is of utmost importance [4]. The best indicator of toric intraocular lens calculation accuracy is the prediction error (PE) of residual astigmatism. Depending on the formula used to calculate toric intraocular lenses, different anatomical parameters are considered [5]. The most important parameters of all formulas are the keratometry values of the cornea since they have the greatest influence on the required toricity of IOL for neutralizing corneal astigmatism [4]. Whereas early formulas solely considered the keratometry values (K) of the anterior corneal surface, state-of-the-art formulas consider the total keratometry (TK) including the values of the anterior and posterior corneal surface. This is due to several studies stating that the posterior corneal surface significantly contributes to the total corneal astigmatism [6], and its consideration thus leads to a superior postoperative prediction accuracy [7].

A vast variety of devices mostly utilize two technologies to acquire corneal keratometry values and biometric ocular data: the rotating Scheimpflug camera and swept-source optical coherence tomography (SS-OCT). One of the most commonly used SS-OCT-based biometric devices is the IOLMaster (Carl Zeiss Meditec AG, Jena, Germany). While a predecessor version of the machine, the IOLMaster 500, was only able to measure the anterior surface of the cornea, the novel IOLMaster 700 also measures the posterior corneal curvature. With the information of the anterior and posterior corneal curvature, the IOLMaster 700 can further calculate the total refractive power of the cornea (total keratometry; TK) [8,9]. An additional feature of the IOLMaster 700 is the ability to calculate intraocular lens power onboard based on the TK values prior to surgery.

With rising numbers of toric intraocular lens implantations, especially in refractive lens exchanges, and the associated extra time effort, surgeons crave an efficient workflow and an IOL power calculation tool to keep preoperative preparation times as short as possible and intraocular lens calculation and implantation as precise as possible. Therefore, the novel cataract workflow EQ Workplace integrated in the FORUM platform (all by Carl Zeiss Meditec AG), featuring the Z CALC intraocular lens formula and calculator, was established to allow a complete digital approach from biometry assessment over the data export to intraoperative toric intraocular lens axis alignment.

To this end, this study determines whether there is a significant saving of time when using the digital cataract workflow EQ Workplace 1.6.0 within the FORUM system for digital data transfer compared to a manual approach of biometry assessment, data export, intraocular lens calculation, and surgery time using the CALLISTO eye and Z ALIGN digital tracking system.

## 2. Materials and Methods

This study is a prospective interventional case series performed at one single tertiary referral center in Munich, Germany (Department of Ophthalmology, University Hospital, LMU Munich, Munich, Germany). Prior to data collection and analysis, approval was obtained by the local institutional review board of the Ludwig Maximilian University (approval number: 19-731). This study complies with the criteria defined in the Declaration of Helsinki. Informed consent was given by all patients included.

The primary goal was to evaluate the saving of time when using the cataract workflow EQ Workplace 1.6.0 within the FORUM system for digital data transfer compared to a manual approach of biometry assessment, intraocular lens calculation, and effective diagnostic and surgery time using the CALLISTO eye and Z ALIGN digital tracking system. Furthermore, this case series will address the accuracy of the Z CALC 2.1.0 intraocular lens calculator (for all: Carl Zeiss Meditec AG, Jena, Germany), embedded in the EQ Workplace, in predicting intraocular lens power and postoperative spherical equivalent. In addition, diverse quality criteria were determined: the reduction of cylinder, the surgically induced astigmatism (SIA), IOL axis misalignment, and the visual outcome.

### 2.1. Patient Characteristics

All patients included either presented with an age-related cataract or a clear crystalline lens and a regular corneal astigmatism of at least one diopter in swept-source optical coherence tomography-based optical biometry using the IOLMaster 700 (Carl Zeiss Meditec AG). The regular corneal astigmatism was confirmed using the central corneal topography acquired with SS-OCT onboard the IOLMaster 700. Exclusion criteria were pseudo-exfoliation syndrome, irregular astigmatism, uveitis, previous vitreoretinal or refractive surgeries, other corneal pathologies, maculopathies, or ocular surface diseases. Both eyes of each study subject underwent clear lens exchange or cataract surgery. One eye received a complete digital approach starting at biometry assessment, whereas the other one underwent a manual approach. For all, phacoemulsification and toric intraocular lens implantation were performed by one experienced surgeon (W.J.M.) using a 2.4 mm clear corneal incision at 90°. To achieve intraoperative toric intraocular lens alignment, the CALLISTO eye and Z ALIGN digital tracking system utilizing a reference image assessed with the IOLMaster 700 prior to surgery, were used. All procedures and study examinations were performed at the Department of Ophthalmology, University Hospital, LMU Munich.

Three months postoperatively, manifest refraction was obtained by subjective refraction according to DIN 58220. Uncorrected and corrected distance visual acuity (UDVA and CDVA) was assessed at six meters (20 ft). Toric IOL axis alignment was evaluated with a slit lamp. To address postoperative SIA, another optical biometry was conducted.

### 2.2. Intraocular Lens Calculation

In all eyes, a monofocal toric (Carl Zeiss AT TORBI 709M, Carl Zeiss Meditec AG) or trifocal toric (Carl Zeiss AT LISA tri toric 939M, Carl Zeiss Meditec AG) intraocular lens with plate haptic design was implanted. The intraocular lens calculations were based on the total keratometry (TK) corneal measurements obtained by optical biometry and performed using the Z CALC intraocular lens calculator in the digital group. In the manual group, IOL were calculated onboard the IOLMaster 700 with the Haigis formula based on the TK values. Surgically induced astigmatism was implemented into the IOL calculation using the surgeon’s reference SIA, calculated with the Warren Hill calculator according to previous standard lens surgeries.

### 2.3. Workflow Steps and Time Measurement Points

The workflow begins with optical biometry and terminates with the end of the surgical intraocular lens implantation. The effective time for all workflow steps were measured whenever the operator or/and the patient were ready for examination/surgery. Table 1 illustrates the time points/intervals of interest (Table 1).

Following optical biometry, patient data was double-checked either onboard the IOLMaster 700 or in the EQ Workplace. Following the IOL calculation explained above, the reference image was either exported via a portable memory drive (USB stick) or directly transferred digitally via the EQ Workplace. After matching the reference image in the manual group, the target IOL axis had to be entered manually. The time of the reference image import as well as the image matching and IOL axis alignment are included in the overall surgery time.

### 2.4. Statistics and Data Analysis

Statistical analysis was performed with the open-source statistics software R (Version 4.1.2; Ross Ihaka and Robert Gentleman, R Core Team, University of Auckland, Auckland, New Zealand). Normality of data was confirmed with the Shapiro–Wilk normality test. To compare the means of time between the manual and digital group, a paired Student’s *t*-test was performed. We considered *p* < 0.01 statistically significant. Results were reported according to the standards for reporting refractive outcomes of intraocular lens-based refractive surgery [10].

## 3. Results

In total, 48 eyes of 24 patients were included in the time measurement study. The mean age was 60 ± 9.8 years (range 42 to 79 years). The proportion of female eyes was 63% (*n* = 30) with 15 patients being female and nine being male. Of those eyes that received a fully digital approach, 18 eyes had a with-the-rule (WTR, axis: 60–120°) astigmatism, five eyes an against-the-rule (ATR, axis: 0–30° or 150–180°) astigmatism and one eye an oblique (axis: 30–60° or 120–150°) astigmatism.

### 3.1. The Digital Approach Using EQ Workplace Saves Diagnostic and Surgical Time

When the surgery was planned, patient identification, data verification in EQ Workplace as well as intraocular lens calculation and reference image export accounted for 48.0 ± 16.1 s (Table 2). Compared to the manual approach of biometric data check and intraocular lens calculation (76.7 ± 12.3 s) as well as the manual export of the reference image to a portable external storage device (26.8 ± 5.5 s), a highly significant saving of time was achieved (*p* < 0.0001).

Prior to surgery, the import of the reference image to the CALLISTO eye-tracking system and matching the same with the patient’s eye took 129.8 ± 18.0 s in the manual and 54.9 ± 9.2 s in the digital group (*p* < 0.0001).

During surgery, intraocular lens alignment was significantly faster using the fully digital approach with 22.8 ± 5.1 s, compared to the manual group (30.7 ± 4.1 s; *p* < 0.0001).

Overall, the complete time of surgery was reduced from 756.5 ± 82.3 s to 667.3 ± 56.3 s using a full EQ Workplace and CALLISTO eye-tracking system approach (*p* < 0.0005; Table 2). The total diagnostic and surgical time came in as 1364.1 ± 202.6 s in the manual group and as 1125.8 ± 183.2 s in the digital group respectively (*p* < 0.001; Table 2).

### 3.2. Reduction of Cylinder Postoperatively

**Figure 1:**
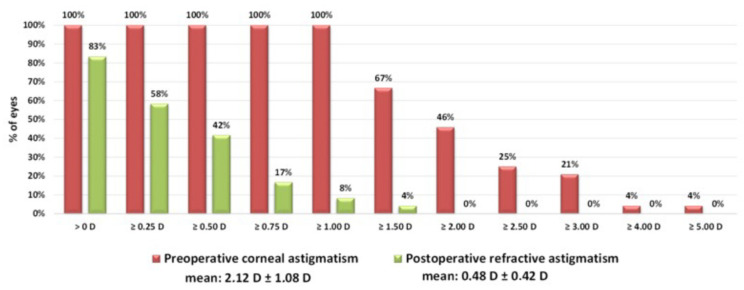
Cumulative histogram of the magnitudes of the preoperative corneal astigmatism and the postoperative refractive astigmatism at the corneal plane in the digital group (*n* = 24).

**Figure 2:**
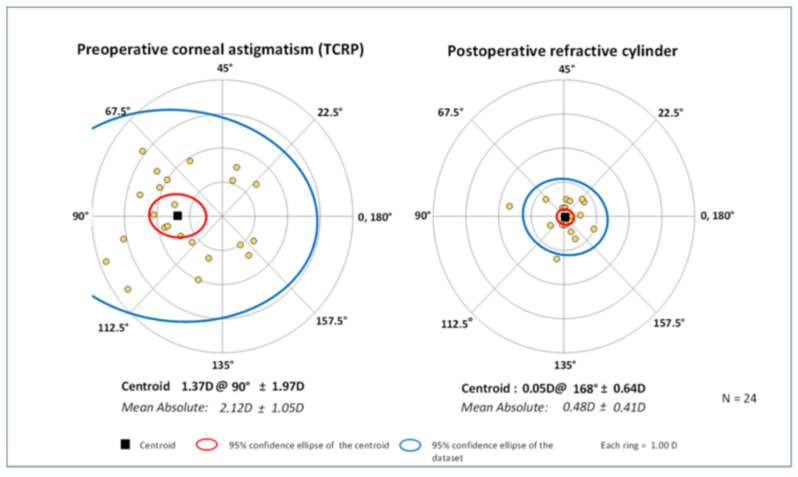
Double-angle plots of preoperative corneal astigmatism (TCRP) and postoperative refractive cylinder in the digital group (*n* = 24).

Following implantation of a toric intraocular lens in the digital group calculated with Z CALC, the mean cylinder of all 24 eyes was reduced significantly from 2.12 ± 1.08 diopters to 0.48 ± 0.42 diopters (*p* = 0.01; Figure 1). Looking at the double-angle plot (Figure 2) and vector analysis, the centroid decreased from 1.37 ± 1.97 diopters at 90° preoperatively to 0.05 ± 0.64 diopters at 168° postoperatively (*p* = 0.03). In total, a residual refractive cylinder of 0.25 diopters or lower could be achieved in 42% of all patients, while in 58% the residual cylinder was 0.50 diopters or lower (Figure 1).

### 3.3. Prediction Error, Visual Outcome and Surgically Induced Astigmatism

The prediction error of spherical equivalent was 0.55 ± 0.43 diopters. Preoperative UDVA was 0.59 ± 0.18 logMAR and 0.4 ± 0.24 logMAR for CDVA. After 3 months, UDVA gained to 0.11 ± 0.09 logMAR and CDVA to 0.05 ± 0.08 logMAR. The mean vector or centroid of the actual postoperative surgically induced astigmatism was 0.21 diopters at 12°. Preoperatively, a SIA of 0.37 diopters at 2° was assumed. Thus, it was slightly overestimated. The SIA prediction error was 0.19 diopters at 9°.

### 3.4. Intraocular Lens Axis Misalignment

Intraocular lens axis was evaluated at the slit lamp. After three months, lens axis misalignment was 2.9 ± 2.7°. In total, 98% of all intraocular lenses were rotated less than 5 degrees. None of the intraocular lenses was re-rotated postoperatively.

## 4. Discussion

Based on our findings, the EQ Workplace within the FORUM platform, the onboard intraocular lens calculator Z CALC, as well as the CALLISTO eye and Z ALIGN digital tracking system offer a convenient and more efficient workflow for lens exchange surgery practice.

The preoperative time to identify the patient, check the biometric data, calculate the toric intraocular lens, and export the reference image for axis alignment was significantly less when the EQ Workplace within the FORUM platform was used. This might be due to the integrated intraocular lens calculator Z CALC and the automatic export of the reference image to the CALLISTO eye system in the operating room. Manually, biometric data must be double-checked on the IOLMaster 700 and the reference image must be transferred via an external storage device from the IOLMaster 700 to the CALLISTO eye system. Therefore, the software-based workflow approach is more efficient for surgeons preoperatively. During surgery, reference image matching and toric intraocular lens alignment is again easier and significantly faster using the digital image-guided approach, as the target axis does not have to be entered manually and the reference image import is basically one finger-tap on the touchscreen compared to the manual import from the USB stick. Thus, it offers surgeons a more comfortable experience prior to surgery. Furthermore, as the overall diagnostic and surgical time is significantly lower, rotation times can be minimized and lead to higher treatment numbers and a better economical outcome. In addition, transcription errors can be minimized when using a digital data transfer.

By increasing numbers of lens exchange surgeries on one day, surgeons and operators tend to mix up patient data and sides. According to a study by ophthalmologists in Israel, surgeons could only identify 73% of their surgical sides (left or right eye) correctly by knowing the patient’s name. This error correlated with the actual number of surgeries performed on one day [11]. In the worst case, this might lead to the implantation of the wrong intraocular lens. Such an error could be minimized by using the EQ Workplace and FORUM platform cataract workflow, where calculated intraocular lenses are directly related to the surgical side and stored in the CALLISTO eye system on the surgical microscope.

The Z CALC intraocular lens calculator and its’ featured intraocular lens calculation formula revealed a prediction error of 0.55 ± 0.43 diopters of spherical equivalent. More than 92% showed a residual cylinder of less than ±1.00 diopters. Those results are in line with recent literature evaluating prediction errors of toric intraocular lenses using novel calculation formulas such as Barrett toric and Kane [12]. Furthermore, toric intraocular lens misalignment was low with a rotation of 2.9 ± 2.7° and no need of any postoperative rotation in any of the subjects. Similar results comparing a manual-marking axis alignment to an image-guided approach corroborate our findings [13,14].

A limitation of our study might be the lack of a direct comparison between a fully manual approach of lens refractive surgery including intraoperative axis alignment by manual marking prior to surgery. This additional detail seemed obsolete as it was investigated before, as mentioned above [13,14]. The time for axis alignment in the digital group in the current study is significantly less statistically compared to the manual group, but, in our opinion, not of clinical relevance as the absolute difference barely accounts for 8 s. We consider those results a surgical bias.

Finally, one must take another issue into consideration: The system relies on a smart digital infrastructure that is costly, and in cases of technical malfunction might lead to severe delays and difficulties in daily lens exchange surgery routine.

To conclude, lens exchange surgery via the EQ Workplace within the FORUM platform is a safe and faster way to acquire and check data as well as to calculate intraocular lenses and align the lens axis intraoperatively compared to a manual approach. Thus, it guarantees a more efficient and economical workflow when performing cataract and lens refractive surgery.

## Figures and Tables

**Table 1:** Workflow steps and points of interest in the manual and digital group.

Manual Group (*n* = 24)	Digital Group (*n* = 24)
Data check	EQ Workplace data check
IOL calculation (IOLMaster 700)	IOL calculation (EQ Workplace)
Export of reference image to USB memory stick	Digital reference image export via FORUM to CALLISTO eye
Import reference image to CALLISTO eye and image matching	Digital reference image import via FORUM to CALLISTO eye and image matching
IOL alignment using CALLISTO eye and Z ALIGN tracking system	
Overall surgery time	
Overall diagnostic + surgery time	

**Table 2:** Time measurements at the time points of interest in the manual and digital group.

Time Point of Interest	Time, Manual Group (*n* = 24; in Seconds)	Time, Digital Group (EQ Workplace) Group (*n* = 24; in Seconds)	Level of Significance
Data check and IOL calculation	76.7 ± 12.3	48.0 ± 16.1	*p* < 0.0001
Reference image export	26.8 ± 5.5		
Reference image import and image matching	129.8 ± 18.0	54.9 ± 9.2	*p* < 0.0001
IOL alignment intraoperatively	30.7 ± 4.1	22.8 ± 5.1	*p* < 0.0001
Surgery time (overall)	756.5 ± 82.3	667.3 ± 56.3	*p* < 0.0005
Diagnostic and surgical time (overall)	1364.1 ± 202.6	1125.8 ± 183.2	*p* < 0.0005

## Data Availability

Not applicable.
